# Platelet-Rich Plasma Combined With Autologous Grafting in the Treatment of Long Bone Delayed Union or Non-union: A Meta-Analysis

**DOI:** 10.3389/fsurg.2021.621559

**Published:** 2021-06-04

**Authors:** Weijun An, Peng Ye, Tao Zhu, Zhizhong Li, Jianbin Sun

**Affiliations:** Department of Trauma Orthopedics, The General Hospital of Ningxia Medical University, Yinchuan, China

**Keywords:** platelet-rich plasma, delayed union, non-union, autologous bone grafting, meta-analysis

## Abstract

**Background:** Platelet-rich plasma (PRP) has been suggested as an emerging treatment for bone defects. However, whether PRP could enhance the therapeutic efficacy of autologous bone grafting for long bone delayed union or non-union remains unknown. A meta-analysis of randomized and non-randomized controlled trials (RCT and NRCT) was performed to summarize current evidence.

**Methods:** Relevant RCTs and NRCTs comparing the influences of autologous bone grafting on healing of long bone delayed union or non-union with and without PRP were obtained by searching PubMed, Embase, Cochrane's Library, China National Knowledge Infrastructure, and WanFang databases from inception to September 10, 2020. A random-effect model was applied to pool the results with the incorporation of the potential heterogeneity. Subgroup analysis according to study design was also performed.

**Results:** Six RCTs and two NRCTs with 420 patients were included. Compared to patients allocated to autologous bone grafting alone, those allocated to combined treatment with PRP and autologous bone grafting were not associated with higher rates of radiographic bone healing [risk ratio (RR): 1.06, 95% confidence interval (CI): 0.99–1.13, *P* = 0.09; *I*^2^ = 24%] or excellent/good posttreatment limb function (RR: 1.14, 95% CI: 0.95–1.37, *P* = 0.37; *I*^2^ = 0%) but was associated with a shorter healing time (mean difference: −1.35 months, 95% CI: −1.86 to −0.84, *P* < 0.001; *I*^2^ = 58%). Subgroup analysis according to study design showed similar results for the above outcomes (*P*-values for subgroup difference all >0.10).

**Conclusions:** Combined treatment with PRP and autologous bone grafting may be effective to accelerate the healing of long bone delayed union or non-union compared to autologous bone grafting alone.

## Introduction

Delayed union and non-union of long bones after fracture are clinically challenging complications, which are observed in up to 40% of patients according to the severity of fracture, location of bones, and damages of vascular tissues, etc. (1–3). Conventional treatment for delayed union and non-union included mechanical fixation and biological stimulation of bone repair, which always involves bone graft to accelerate the healing of the defect (4–7). Among these treatment strategies, autologous bone grafting has become one of the most important treatments for delayed union and non-union of long bones, which should be considered 4~6 months after fracture, primarily because of its osteogenic efficacy (8, 9). However, for some cases, healing of delayed union and non-union of long bones remains slow despite autologous bone grafting (10, 11). Therefore, combined treatments to enhance the repair efficacy of autologous bone grafting on bone defects are still urgently needed.

Platelet-rich plasma (PRP) is a concentrated preparation of autologous plasma from the patient's own peripheral blood (12). Previous studies demonstrated that PRP is enriched with various growth factors and cytokines that could augment the natural healing process of injured bones and soft tissues (13–15). Accordingly, PRP has been well-proposed as a potential treatment for some osteoarticular diseases, such as osteoarthritis (16, 17). Besides, accumulating case studies or case series suggest that PRP injection may improve the healing of bone delayed union and non-union (18–21). However, it remains unknown whether combined treatment with PRP and autologous bone grafting could improve the healing of delayed union and non-union of long bones compared to treatment of autologous bone grafting alone (22). In view of the inconsistent results in previous pilot controlled studies (23–30), we aimed to perform a meta-analysis to systematically evaluate whether PRP could enhance the therapeutic efficacy of autologous bone grafting for long bone delayed union or non-union.

## Methods

This systematic review and meta-analysis were designed and performed in accordance with the PRISMA (Preferred Reporting Items for Systematic Reviews and Meta-Analyses) statement (31) and the Cochrane Handbook guidelines (32).

### Search Strategy

PubMed, Embase, Cochrane's Library (Cochrane Center Register of Controlled Trials), China National Knowledge Infrastructure (CNKI, http://www.cnki.net/), and WanFang (http://www.wanfangdata.com.cn/) electronic databases were systematically searched for relevant studies using a combination of the following terms: (1) “platelet-rich plasma” OR “PRP”; (2) “autologous bone graft” OR (“bone” AND “graft”); and (3) “non-union” OR “non-union” OR “non union” OR “pseudoarthrosis” OR “delayed union” OR “ununited” OR “atrophic bone.” The search was limited to clinical studies in humans. We also manually analyzed reference lists of the original and review articles. The final database search was performed on September 10, 2020.

### Study Selection

Studies were included if they met the following criteria: (1) published as full-length articles in English or Chinese; (2) reported as randomized controlled trials (RCTs) or non-randomized controlled trials (NRCTs) with a parallel design; (3) patients aged 18 years and older with delayed union or non-union of tibia, fibula, femur, ulna, radius, or humerus more than 4 or 6 months after fracture were included (1); (4) patients were randomly or non-randomly allocated to a treatment group of PRP combined with autologous bone graft and a control group of autologous bone graft alone; and (5) reported at least one of the following outcomes: patients with postoperative healing of delayed union or non-union, as evidenced by radiographic findings in each group, mean healing time, or patients with excellent/good posttreatment limb function in each group. Reviews, studies including children or neonates, preclinical studies, case reports or case series studies, and repeated reports were excluded. Definition of radiographic healing was consistent with the criteria used among the included studies, which typically involved the formation of bridging callus on radiographic views.

### Data Extraction and Quality Assessment

Two authors performed the literature search, data extraction, and quality assessment independently in accordance with the inclusion criteria. Discrepancies were resolved by consensus. Extracted data included the location of the study, study design characteristics (RCTs or NRCTs), characteristics of delayed union or non-union (location and time after fracture), patient characteristics (number, age, and sex), treatments in intervention and control groups, and follow-up durations. We applied the seven domains of the Cochrane's Risk of Bias Tool to evaluate the quality of the included RCTs (32), which include criteria regarding random sequence generation, allocation concealment, blinding of participants and personnel, blinding of outcome assessors, incomplete outcome data, selective outcome reporting, and other potential threats to validity. Quality of NRCTs were evaluated with the ROBINS-I checklist (33), which were judged for confounding bias, selection bias, bias in classification of interventions, bias in deviation from intended interventions, bias due to missing data, bias in measurement of outcome, and bias in selection of the reported results.

### Statistical Analysis

Outcomes of categorized variables (ratios of patients with postoperative bone healing and excellent/good posttreatment limb function) were presented as risk ratio (RR) with 95% confidence intervals (CIs), while outcome of continuous variable (healing time after surgery) was presented as mean difference (MD) and 95% CI. Cochrane's Q test was applied to evaluate the heterogeneity among the included studies, and significant heterogeneity was considered for *P* < 0.10 (34). The I^2^ statistic, which describes the percentage of total variation across studies that is due to heterogeneity rather than chance (34), was also calculated. An I^2^ > 50% indicated significant heterogeneity. Pooled analyses were calculated using a random-effect model because this method could incorporate the influence of potential heterogeneity and retrieve a more generalized result (32). Subgroup analyses comparing the results in RCTs and NRCTs were also performed. Sensitivity analysis by omitting one study at a time was used to evaluate the robustness of the results (32). Potential publication bias was firstly evaluated by visual inspection of funnel plots and then evaluated with Egger's regression asymmetry test (35) if at least 10 datasets were included for the meta-analysis. *P*-values were two-tailed, and statistical significance was set at 0.05. We used RevMan (Version 5.1; Cochrane, Oxford, UK) and Stata software (Version 12.0; Stata, College Station, TX) for the statistical analyses.

## Results

### Search Results

**Figure 1:**
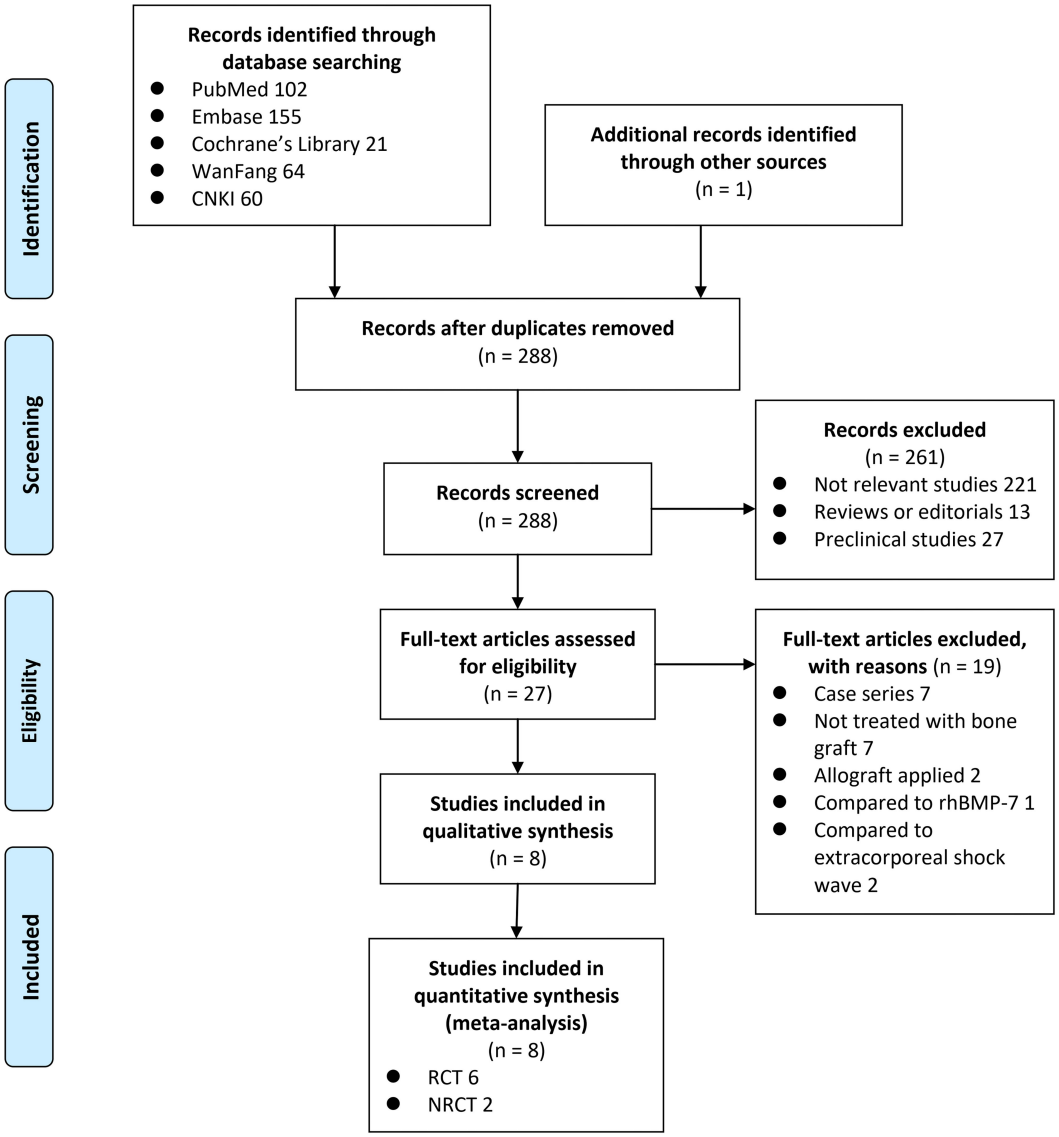
Flowchart of literature search.

A total of 403 articles were identified through the database search; after exclusion of duplicates, 288 articles were screened. Among them, 261 articles were subsequently excluded based on title and abstract screening mainly because they were not relevant to the purpose of the study. Of the 27 potentially relevant articles, 19 were further excluded *via* full-text review based on reasons listed in Figure 1. Finally, eight studies [six RCTs (23, 24, 26–29) and two NRCTs (25, 30)] were included.

### Study Characteristics

The characteristics of the included RCTs and NRCTs are summarized in Tables 1, 2. Overall, six RCTs (23, 24, 26–29) and two NRCTs (25, 30) with a total of 420 adult patients with long bone delayed union or non-union were included. Three of the included studies were published in English (24, 27, 30) and performed in Iran, Mexico, and Iraq, respectively. The other five studies were performed in China and published in Chinese (23, 25, 26, 28, 29). All of the studies included patients with long bone aseptic non-union except for one study that enrolled patients with aseptic humeral shaft delayed union (27). The number of the included patients ranged from 16 to 92 among each study, and the mean ages varied between 26 and 38 years. The mean time from fracture to the current treatment of patients included in each study varied from 5 to 18 months. For patients allocated to the treatment group, combined treatment with PRP and autologous bone graft was applied; while for those allocated to the control group, autologous bone graft alone was performed. The processes of PRP preparation were described in Table 2, which involved a two-step centrifugation method with different g-forces and times used in centrifugation. Autologous bone graft was performed *via* autologous ilium in seven of the included studies (23–27, 29, 30), while the other one did not specify the origin of autologous bone graft (28). The follow-up durations varied from 9 to 25 months after surgery. For all of the included studies, bone healing was defined as radiographic healing, which typically involved the formation of bridging callus on radiographic views (Table 2).

**Table 1:** Study design, patient characteristics, and details of interventions of the included studies.

**Study**	**Country**	**Design**	**Characteristics of delayed union or non-union**	**Number of patients**	**Mean age (years)**	**Male (%)**	**Mean time since fracture (months)**	**Intervention**	**Control**
Xu et al. (23)	China	R	Aseptic tibial non-union	60	38	63	11	PRP + autologous ilium	Autologous ilium
Ghaffarpasand et al. (24)	Iran	R, DB, PC	Aseptic long bone (femur, tibia, humerus, ulna) non-union	75	26	85	18	PRP + autologous iliac crest graft	Saline+ autologous iliac crest graft
Sun et al. (25)	China	NRCT	Aseptic humerus non-union	27	36	59	12	PRP + autologous iliac crest graft	Autologous iliac crest graft
Zhang et al. (26)	China	R	Aseptic long bone (femur, tibia, humerus, ulna) non-union	56	32	70	8	PRP + autologous iliac crest graft	Autologous iliac crest graft
Zheng et al. (29)	China	R	Aseptic humerus non-union	62	36	63	12	PRP + autologous iliac crest graft	Autologous iliac crest graft
Acosta-Olivo et al. (27)	Mexico	R, SB	Aseptic humeral shaft delayed union	16	38	81	5	PRP + autologous iliac crest graft	Autologous iliac crest graft
Zhao et al. (28)	China	R	Atrophic femoral shaft non-union	92	NR	NR	9	PRP + autologous bone graft	Autologous bone graft
Majeed et al. (30)	Iraq	NRCT	Aseptic distal tibia atrophic non-union	32	35	84	NR	PRP + autologous iliac crest graft	Autologous iliac crest graft

*R, randomized; DR, double-blinded; PC, placebo-controlled; SB, single-blinded; NRCT, non-randomized controlled trial; NR, not reported; PRP, platelet-rich plasma*.

**Table 2:** Protocols for PRP preparation and definitions of fracture healing.

**Study**	**Details of PRP preparation**	**Details of PRP administration**	**Follow-up duration (months)**	**Definitions of healing**
Xu et al. (23)	Venous blood 200 ml, anticoagulated with acid-citrate dextrose, firstly centrifuged at 3,740 rpm for 14 min to separate RBC, then centrifuged at 1,200 rpm for 14 min to generate PRP	PRP 20 ml (8–10 times PLT compared to native blood), activated by calcium chloride, and applied with autologous ilium graft to the non-healing area	22	Radiographic union evaluated by Score of Lane and Sandhu
Ghaffarpasand et al. (24)	Prepared with Gravitational Platelet Separation System; Venous blood 54 ml, anticoagulated with acid-citrate dextrose, firstly centrifuged at 3,200 rpm for 15 min to separate RBC, and then removing platelet-poor plasma to generate PRP	PRP 5–6 ml (5.2–5.8 times PLT compared to native blood) and applied with autologous iliac crest graft	9	Radiological union defined as the presence of bridging callus on at least 3/4 cortices on the AP and lateral radiographic views
Sun et al. (25)	Venous blood 200 ml, anticoagulated with acid-citrate dextrose, firstly centrifuged at 2,500 rpm for 10 min to separate RBC, then centrifuged at 2,500 rpm for 10 min to generate PRP	PRP 6–8 ml, activated by thrombin, and applied with autologous iliac crest graft	25	Radiological union
Zhang et al. (26)	Venous blood 80 ml, anticoagulated with sodium citrate, firstly centrifuged at 100 g for 20 min to separate RBC, then then centrifuged at 250 g for 10 min to generate PRP	PRP 10 ml (4–5 times PLT compared to native blood) and applied with autologous iliac crest graft	NR	Radiological union evaluated by scores of bridging callus formation according to the standard of local institution
Zheng et al. (29)	Venous blood 200 ml, firstly centrifuged at 2,500 rpm for 10 min to separate RBC, then centrifuged at 2,500 rpm for 10 min to generate PRP	PRP 6–8 ml, activated by thrombin, and applied with autologous iliac crest graft	NR	Radiological union
Acosta-Olivo et al. (27)	Venous blood 54 ml, anticoagulated with sodium citrate, centrifuged at 1,800 rpm for 5 min to separate RBC, then centrifuged at 3,200 rpm for 3 min to generate PRP	PRP 12 ml, activated by calcium gluconate, and applied with autologous iliac crest graft	9	Radiological union evaluated by extent of new bone formation on the AP and lateral radiographic views
Zhao et al. (28)	Venous blood 30 ml, anticoagulated with sodium citrate, centrifuged at 200 g for 10 min to separate RBC, then centrifuged at 200 g for 10 min to generate PRP	PRP 5 ml, activated by thrombin, and applied with autologous bone graft	9	Radiological union
Majeed et al. (30)	Venous blood 54 ml, after adding anticoagulant, then centrifuged twice to generate PRP	PRP applied with autologous iliac crest graft	9	Radiological union evaluated by callus formation on the AP and lateral radiographic views

*PRP, platelet-rich plasma; PLT, platelet count; NR, not reported; AP, anteroposterior*.

### Data Quality

The details of risks of biases of the RCTs and NRCTs according to the Cochrane assessment tool and ROBINS-I checklist are shown in Table 3. For RCTs, two studies were double-blind (24) and single-blind (27), respectively, while the other four were open-label (23, 26, 28, 29). Details of random sequence generation were reported in four studies (24, 26, 28, 29), and details of allocation concealment were reported in only one study (27). The overall quality score for the included RCTs varied between 3 and 6, indicating moderate to good study quality. For the two NRCTs, the overall quality scores evaluated by the ROBINS-I checklist were 7 and 6, respectively, suggesting overall good study quality.

**Table 3:** Details of study quality evaluation.

**Study**	**Random sequence generation**	**Allocation concealment**	**Blinding of participants**	**Blinding of outcome assessment**	**Incomplete outcome data addressed**	**Selective reporting**	**Other sources of bias**	**Total**
**RCT**								
Xu et al. (23)	Unclear	Unclear	Unclear	Unclear	Low	Low	Low	3
Ghaffarpasand et al. (24)	Low	Unclear	Low	Low	Low	Low	Low	6
Zhang et al. (26)	Low	Unclear	Unclear	Unclear	Low	Low	Low	4
Zheng et al. (29)	Low	Unclear	Unclear	Unclear	Low	Low	Low	4
Acosta-Olivo et al. (27)	Unclear	Low	Low	Unclear	Low	Low	Low	5
Zhao et al. (28)	Low	Unclear	High	High	Low	Low	Low	4
**Study**	**Bias due to confounding**	**Bias in selection of participants into the study**	**Bias in classification of interventions**	**Bias due to deviations from intended interventions**	**Bias due to missing data**	**Bias in measurement of outcomes**	**Bias in selection of the reported result**	**Total**
**NRCT**								
Sun et al. (25)	Low	Low	Low	Low	Low	Low	Low	7
Majeed et al. (30)	Low	Low	Low	Low	Low	Unclear	Low	6

*RCT, randomized controlled trial; NRCT, non-randomized controlled trial*.

### Meta-Analysis of the Ratios of Patients With Postoperative Healing

**Figure 2:**
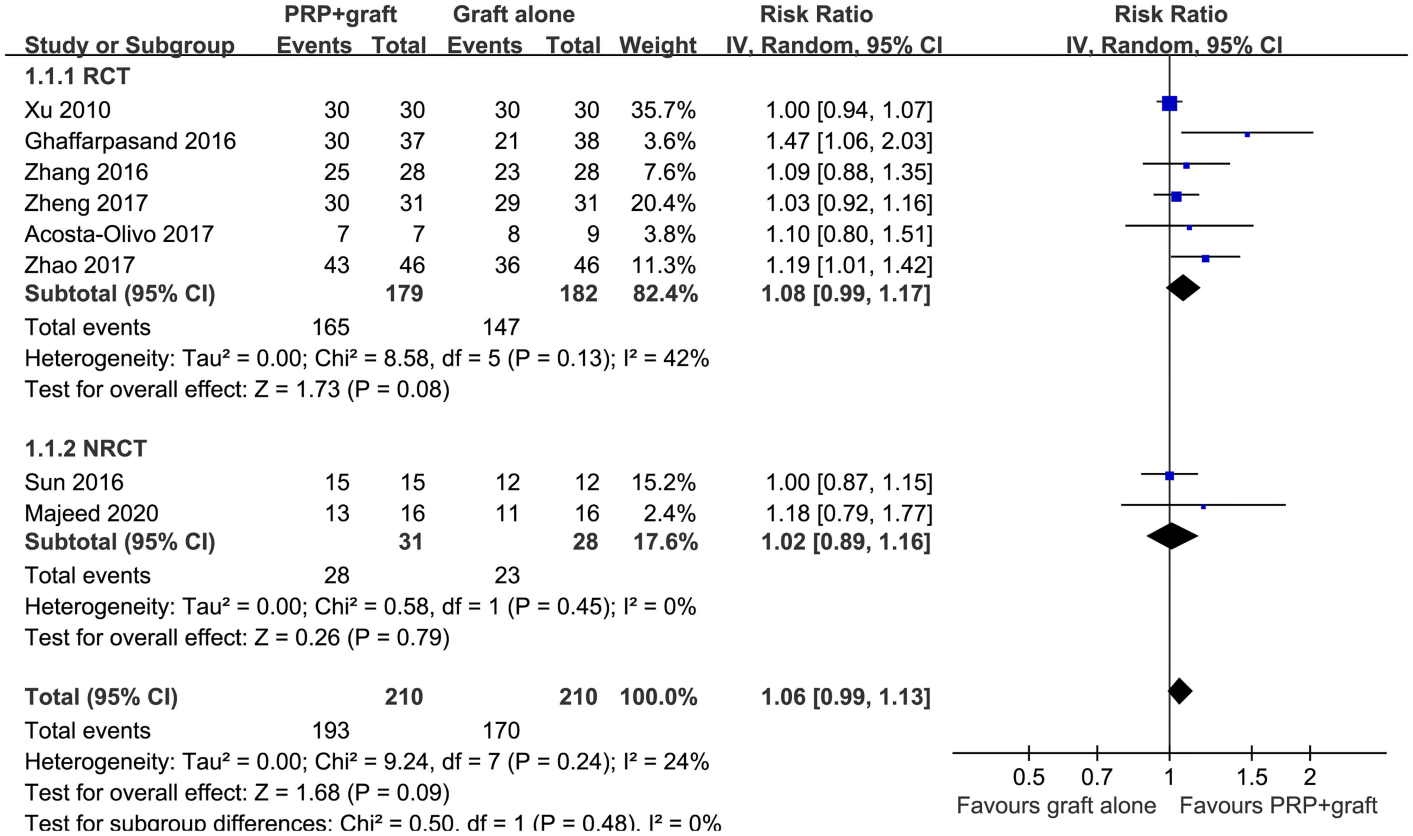
Forest plots for the meta-analysis of ratios of patients with bone healing in patients with long bone delayed union or non-union stratified by study design.

All of the included studies reported the ratios of patients with postoperative healing in patients with long bone delayed union or non-union, with mild heterogeneity (*P* for Cochrane's Q test = 0.24, *I*^2^ = 24%). Pooled results with a random-effect model showed that compared to autologous bone grafting alone, combined treatment with PRP and autologous bone grafting was not associated with a higher ratio of patients with bone healing after treatment (RR: 1.06, 95% CI: 0.99–1.13, *P* = 0.09; Figure 2). Sensitivity by excluding one study including patients with delayed union showed similar results (RR: 1.06, 95% CI: 0.99–1.14, *P* = 0.11; *I*^2^ = 34%). Subgroup analysis showed that the results were consistent in RCTs and NRCTs (*P* for subgroup difference = 0.48; Figure 2).

### Meta-Analysis of Average Healing Time

**Figure 3:**
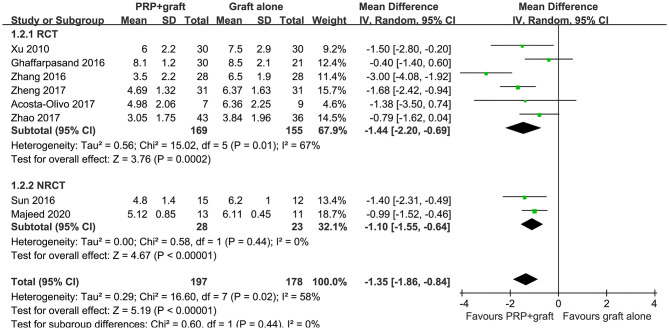
Forest plots for the meta-analysis of average healing time in patients with long bone delayed union or non-union stratified by study design.

The outcome of healing time in patients with long bone delayed union or non-union were also reported in all of the eight included studies, with significant heterogeneity (*P* for Cochrane's Q test = 0.02, *I*^2^ = 58%). The average time to healing varied from 3 to 8 months for patients from both groups among the included studies. Pooled results with a random-effect model showed that combined treatment with PRP and autologous bone grafting was associated with shorter healing time (MD: −1.35 months, 95% CI: −1.86 to −0.84, *P* < 0.001; Figure 3). Sensitivity by excluding one study including patients with delayed union did not significantly change the results (MD: −1.35 months, 95% CI: −1.89 to −0.81, *P* < 0.001; *I*^2^ = 64%). Subgroup analysis showed consistent results in RCTs and NRCTs (*P* for subgroup difference = 0.44; Figure 3).

### Meta-Analysis of the Ratios of Patients With Excellent/Good Posttreatment Limb Function

**Figure 4:**
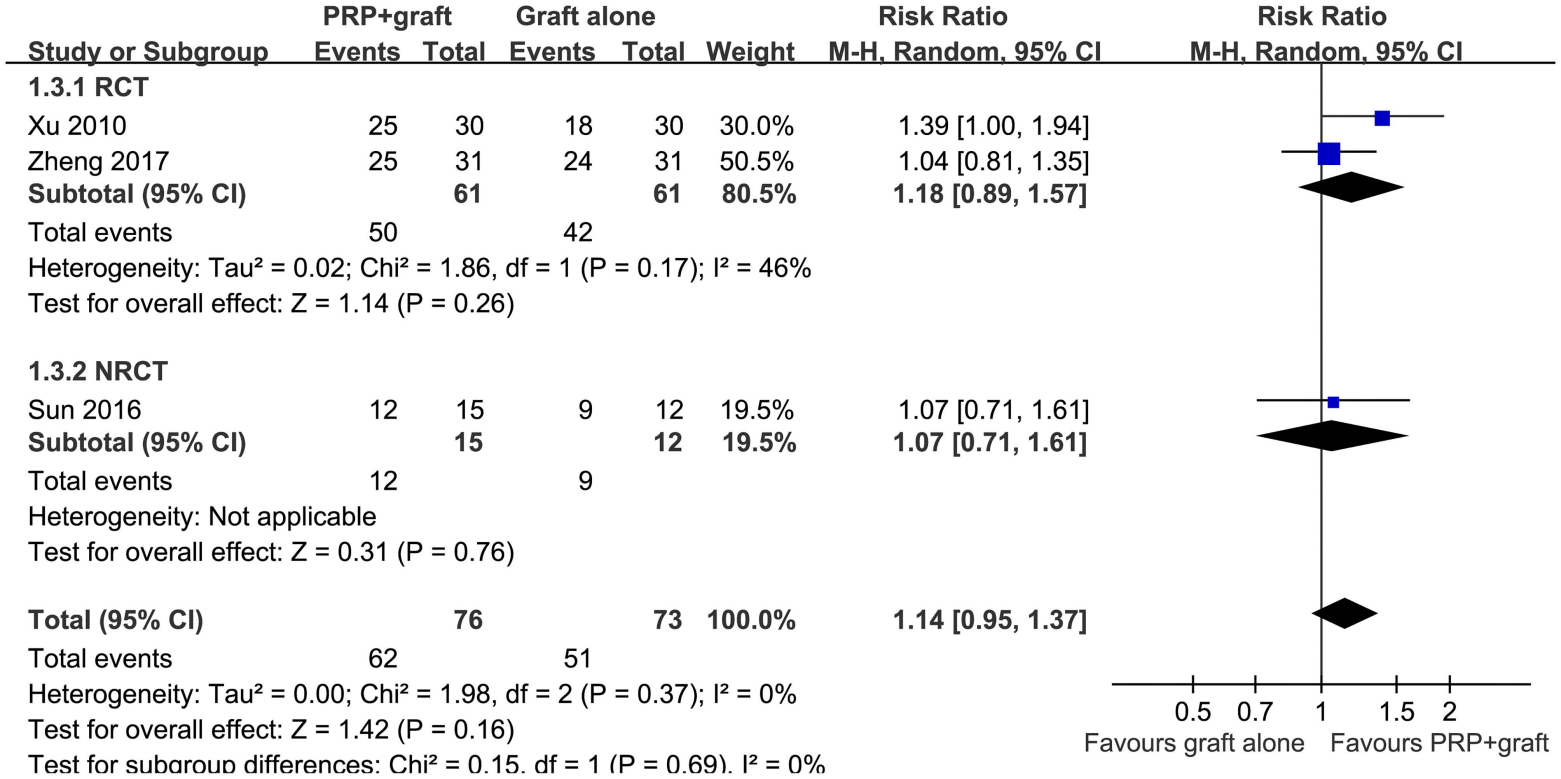
Forest plots for the meta-analysis of ratios of patients with excellent/good posttreatment limb function in patients with long bone non-union stratified by study design.

Pooled results of three studies (23, 25, 29), all including patients with long bone non-union, showed that ratios of patients with excellent/good posttreatment limb function were similar between two treatments (RR: 1.14, 95% CI: 0.95–1.37, *P* = 0.37; *I*^2^ = 0%; Figure 4). Subgroup analysis showed consistent results in RCTs and NRCTs (*P* for subgroup difference = 0.69; Figure 4).

### Publication Bias

**Figure 5:**
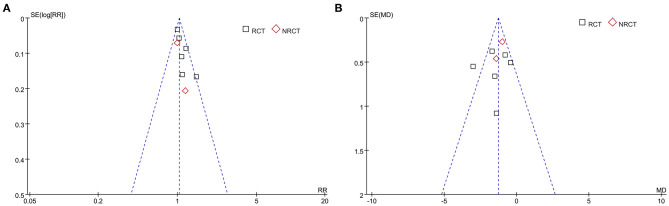
Funnel plots for the meta-analysis of ratios of patients with postoperative healing and average healing time in patients with long bone delayed union or non-union. **(A)** Ratios of patients with postoperative healing. **(B)** Average healing time.

The funnel plots for the meta-analyses of ratios of patients with postoperative healing and average healing time in patients with long bone delayed union or non-union were symmetrical on visual inspection, suggesting low-risk publication bias (Figures 5A,B). Egger's regression tests were not performed since <10 datasets were included for these outcomes. The publication bias underlying the meta-analysis of ratio of patients with excellent/good posttreatment limb function was difficult to estimate, since only three studies were included for the outcome.

## Discussion

In this meta-analysis, by pooling the results of available randomized and non-randomized studies, we found that compared to autologous bone grafting alone, combined treatment with PRP and autologous bone grafting was associated with shorter average healing time for delayed union and non-union of long bones after fracture, although the rates of patients with bone healing and excellent/good posttreatment limb function were similar between patients who received two treatments. Results of subgroup analysis showed similar results in RCTs and NRCTs. Taken together, this meta-analysis indicated that combined treatment with PRP and autologous bone grafting may accelerate the healing of long bone delayed union or non-union compared to autologous bone grafting alone, and the clinical relevance of the findings should be evaluated in future studies.

To the best of our knowledge, our study is the first meta-analysis comparing PRP and autologous bone grafting vs. autologous bone grafting alone in patients with long bone delayed union or non-union. We found that compared to autologous bone grafting alone, combination with PRP could shorten the mean healing time of long bone non-union by 1.35 months. This finding may be clinically relevant, since early healing of long bone non-union after fracture has been associated with improved functional and clinical outcomes of the patients (36, 37). Besides, a significantly shortened healing time of long bone non-union is probably associated with less medical expenses of the patients and the health care system (38). Moreover, PRP could be conveniently obtained and prepared based on autologous blood of the patients and could be easily applied during the surgery of autologous bone grafting, which suggests the feasibility of the combined treatment in clinical practice (39).

As for the outcome of the ratio of patients with bone healing at the end of the follow-up, although the result of the meta-analysis was non-significant, a trend of superiority of the combined treatment with PRP and autologous bone grafting to autologous bone grafting alone could be also observed (*P* = 0.09 for meta-analysis of all studies and *P* = 0.08 for meta-analysis of RCTs). In addition, this meta-analysis failed to show that the combined treatment was associated with better limb functional outcome compared with autologous bone grafting alone. However, only three studies were available in the meta-analysis with this outcome, and future studies are needed for further evaluation.

The exact mechanisms underlying the benefits of PRP on healing of bone defects remain not fully understood and probably are multifactorial (40). Early studies showed that the pro-inflammatory cytokines secreted by platelets could regulate the inflammatory phase of bone healing, including interleukin-1 beta (IL-1β), CD40L, and chemokines, etc. (41). Subsequent studies suggested that besides inflammatory cytokines, PRP is also enriched in various platelet-secreted growth factors, which are also involved in the process of bone healing and related soft tissue repair, such as platelet-derived growth factor (PDGF), vascular endothelial growth factor (VEGF), transforming growth factor-beta (TGF-β), fibroblast growth factor (FGF), insulin-like growth factor (IGF), and epidermal growth factor (EGF) (13). Some of these growth factors play a direct role in promoting the healing of bone defects (42). For example, activated PDGF was reported to attach to transmembrane receptors on osteoblasts, osteoclasts, chondrocytes, fibroblasts, and macrophages to stimulate mitogenesis, angiogenesis, bone remodeling, and phagocytosis of damaged tissue during fracture healing (43). Recent evidence also suggests that growth factors derived from PRP could interact with mesenchymal stem cells, which synergistically regulate the healing of bone defects (44). The exact mechanisms and key pathways underlying the benefits of combined PRP and autologous bone grafting on healing of long bone non-union deserve further studies.

Some limitations of the current meta-analysis should be considered when the results are interpreted. Firstly, the numbers of available studies and the included patients for some outcomes were limited. For example, meta-analysis of limb function after surgery only involved three studies, the results of which should be validated in large-scale RCTs. Moreover, quality scores of the included RCTs were moderate, some of which did not specify group allocation or blinding. Besides, both RCTs and NRCTs were included in the current meta-analysis, although subgroup analysis according to study design was performed. Accordingly, high-quality large-scale RCTs with adequate statistical power are warranted to verify our findings. In addition, the optimal PRP formulation and the protocols for PRP administration during the surgery remain unknown, and the influences of these factors on the outcomes were not evaluated because most of the included studies did not provide adequate details related to PRP preparation. Furthermore, the pathophysiological mechanisms of long bone non-union may be different according to the location of the fracture. The influence of fracture location on the efficacy of PRP combined with autologous grafting on non-union healing remains unknown, since limited studies were available for such an analysis. Moreover, definitions of bone healing varied among the included studies, which may contribute to the clinical heterogeneity of the included studies. In addition, radiological healing was applied to evaluate the outcome in all of the studies. It remains unclear whether the results were consistent if outcomes based on clinical healing were applied. Finally, the influence of other clinical characteristics, such as fracture type, mechanical fixation, amount and mode of autologous bone transplantation, and follow-up duration on the outcomes of the meta-analysis should also be evaluated in future large-scale RCTs.

In conclusion, results of this meta-analysis suggest that combined treatment with PRP and autologous bone grafting may accelerate the healing of long bone delayed union or non-union compared to autologous bone grafting alone. Future high-quality RCTs are needed to confirm these findings and to explore the optimal formulation of PRP used for accelerating the healing of long bone non-union.

## Data Availability Statement

The original contributions presented in the study are included in the article/supplementary material, further inquiries can be directed to the corresponding author.

## Author Contributions

WA and JS designed the study. WA and PY performed database search and data extraction. PY and TZ performed quality assessment of the included studies. WA, PY, TZ, and ZL performed statistical analysis and result interpretation. WA and PY drafted the manuscript. All authors critically revised the manuscript and approved its submission.

## Conflict of Interest

The authors declare that the research was conducted in the absence of any commercial or financial relationships that could be construed as a potential conflict of interest.
